# Methadone Withdrawal-Related Psychosis in a Patient With Hormone-Dependent Breast Cancer: A Case Report

**DOI:** 10.7759/cureus.51240

**Published:** 2023-12-28

**Authors:** Mauro Pinho, Daniela O Martins, Francisco Coutinho

**Affiliations:** 1 Acute Psychiatry Service Unit, Centro Hospitalar Universitário de Santo António - Hospital de Magalhães Lemos, Porto, PRT

**Keywords:** aripiprazole, prolactin, breast cancer, addiction, opioid, methadone withdrawal, psychosis

## Abstract

Methadone withdrawal usually presents as a classical opiate withdrawal syndrome, including symptoms such as restlessness, pupillary dilation, sweating, insomnia, irritability, sneezing, nausea, vomiting, and diarrhea. It rarely manifests as psychosis. Here, we discuss the case of a 43-year-old female with a history of long-term methadone use who presented with first-episode psychosis during methadone down-titration. She exhibited persecutory delusions and auditory hallucinations, unrelated to classical opiate withdrawal symptoms. Medical tests were unremarkable. The patient was diagnosed with first-episode psychosis and was involuntarily admitted to our psychiatric hospital. As she suffered from hormone-dependent breast cancer and presented paliperidone-induced hyperprolactinemia, we switched this drug to aripiprazole, a prolactin-sparing antipsychotic. Her psychotic symptoms remitted in six weeks, with no reintroduction of methadone. It remains unclear whether this presentation is attributable to a rare manifestation of withdrawal or methadone’s antipsychotic properties, masking an underlying psychotic disorder. This case contributes to understanding psychosis emergence post-opioid withdrawal, underscoring the need for further investigation into withdrawal-related psychosis and opioid antipsychotic properties. It also prompts the discussion of antipsychotic treatment in patients with comorbid breast cancer, while evidence about hyperprolactinemia as a risk factor for breast cancer remains conflicting.

## Introduction

Methadone is a typical agonist of the opiate μ receptor, prescribed as an opioid substitution treatment [1]. Methadone withdrawal usually presents as a classical opiate withdrawal syndrome, including symptoms such as restlessness, pupillary dilation, sweating, insomnia, irritability, sneezing, nausea, vomiting, and diarrhea [2]. Untreated methadone withdrawal typically reaches its peak between four and six days after the last dose, and symptoms persist for 10 to 12 days [3]. There are a few case reports of psychosis as a manifestation of opioid withdrawal, including methadone [2]. In these cases, patients presented with psychotic symptoms such as persecutory delusions and auditory hallucinations, not associated with other typical symptoms of methadone withdrawal and unrelated to an acute confusional state. In one case, symptoms resolved spontaneously; some cases were treated with methadone reintroduction, whereas others also required antipsychotic treatment [1,2,4,5].

Some case reports suggest the use of methadone as beneficial in the control of psychotic symptoms in patients with schizophrenia. However, there are no controlled studies of opiate use in the treatment of psychosis. Additionally, the mechanism of action of methadone as an antipsychotic is not clear [6-8]. Here, we present a case of first-episode psychosis starting during methadone down-titration.

This article was previously presented as an abstract at the XV National Congress of Psychiatry, in Albufeira (Portugal), on November 19, 2021.

## Case presentation

A Portuguese 43-year-old female patient was taken to the psychiatric emergency room by the authorities because of violent behavior directed against her neighbors. Her chief complaint was of being a victim of “a game where everyone is connected.” The patient’s symptoms began one month before when she started to suspect her neighbors had started “a game” to help her with financial issues. She claimed that, once the town mayor found out, he managed to put everyone against her and her neighbors started prosecuting her: “I started understanding everything … the words they were saying … it was all a game against me, just a play to get me.” She started hearing people talking about her with contempt in the street, shouting and insulting her. Angry, she started banging on everyone’s doors and cars during the night, as she couldn’t sleep, so the neighbors called for the authorities. According to her sister, “she is not acting normal … she is hearing voices, talks to herself and tells me people are talking about her in the street.”

The patient had a psychiatry history of heroin use, beginning in adolescence, and had been followed up at a center for addiction since then. She was on a long-term maintenance treatment with methadone 60 mg for the last 15 years. As she had not used heroin for the last four years, her methadone prescription was down-titrated in the last two months at a rate of about 5-10 mg per week. Nine days before admission, she stopped taking methadone. The patient also had a medical record of a recently diagnosed hormone-dependent breast cancer, with no metastasis, and was waiting for an already scheduled mastectomy. She was also diagnosed with autoimmune thyroiditis, under treatment with levothyroxine 0.1 mg. She had no family history of mental disorders.

At the mental status examination, the patient was alert and oriented, with a normal mood, persecutory delusion, auditory hallucinations, and no insight into her condition. She presented no classical opiate withdrawal syndrome, including symptoms such as pupillary dilation, sweating, sneezing, nausea, vomiting, or diarrhea.

Her blood test and brain CT scan were normal, including her thyroid function. Her urine drug test was negative for opioids, cocaine, cannabinoids, amphetamines, and phencyclidine.

She was diagnosed with first-episode psychosis and was involuntarily admitted as an inpatient to our psychiatric hospital.

We began treatment with paliperidone 6 mg, but this drug induced hyperprolactinemia (99.9 ng/mL of prolactin). As the patient suffered from hormone-dependent breast cancer, we switched paliperidone for aripiprazole, as this antipsychotic is prolactin-sparing [9]. The patient’s prolactin levels were monitored, returning to the normal range (from 99.9 to 24.1 ng/mL). We did not reintroduce methadone, as the patient preferred not to. The patient’s response to treatment was slow and gradual, but her psychotic symptoms were successfully remitted at six weeks under aripiprazole 30 mg.

## Discussion

This patient had no prior history of psychosis and her psychosis onset was related to the down-titration of methadone, which aggravated after full discontinuation of this drug, resulting in her involuntary admission. Although she presented no classical opiate abstinence syndrome, the temporal relationship between the two events suggests there might be a causality.

It is also possible that the patient suffered from an underlying psychotic disorder that was successfully being treated with methadone, given the opioid’s suggested antipsychotic properties [6-8,10]. Arguments favoring this hypothesis include the fact that the patient’s psychotic symptoms took several weeks to remit, lasting longer than typical methadone withdrawal syndrome [3], with treatment requiring high aripiprazole dosages.

Opioid’s antipsychotic mechanism of action is not clear. However, opioid agonists may have a modulatory effect on dopaminergic functions, as there is evidence that they effectively alter dopamine release, dopamine reuptake, and dopamine metabolism in the striatum and substantia nigra [10]. There are several case reports of methadone use as a successful add-on treatment in patients suffering from schizophrenia, manic episodes, and acute psychosis [6-8].

In a systematic review, Lozano-López et al. (2021) found seven case reports in which there was a temporal relationship between the withdrawal of several opiates, including methadone, and the onset of psychotic symptoms, with symptoms responding to the reintroduction of the opioid [2]. However, other case reports also documented spontaneous remission of psychotic symptoms or the necessity of antipsychotic drugs [1,4,5].

Our patient suffered from comorbid hormone-dependent breast cancer. There are conflicting data on whether hyperprolactinemia is associated with an increased risk of breast cancer in women [11]. In animals, there is evidence that increased prolactin levels can cause malignant transformation of breast tissue and promote tumor growth in induced mammary malignancies [12]. Wang et al. (2001) found that dopamine receptor antagonists conferred a small but significant risk of breast cancer in a retrospective cohort study conducted among 52,819 women exposed to these drugs [13]. Therefore, it is reasonable to avoid prolactin-elevating drugs in these patients. Aripiprazole, as a prolactin-sparing antipsychotic, might be a safe choice [9].

## Conclusions

This case report adds to several others showing psychosis onset after opioid withdrawal. It remains unclear whether this presentation is attributable to a rare manifestation of withdrawal or methadone’s antipsychotic properties, masking an underlying psychotic disorder. This case contributes to understanding psychosis emergence post-opioid withdrawal, underscoring the need for further investigation into withdrawal-related psychosis and opioid antipsychotic properties. In addition, prompts the discussion of antipsychotic treatment in patients with comorbid breast cancer, while evidence about hyperprolactinemia as a risk factor for breast cancer remains conflicting.
